# Comparison of the importance and observance of the patient's bill of rights from the perspectives of patients and personnel of hospitals in Kerman

**DOI:** 10.18502/jmehm.v13i5.4070

**Published:** 2020-06-02

**Authors:** Mahlagha Dehghan, Roghayeh Mehdipour-Rabori, Masoud Rayani, Mohammad Ali Zakeri, Mina Mobasher, Maryam Iranmanesh, Narges Rezai

**Affiliations:** 1 *Assistant Professor, Nursing Research Center, Kerman University of Medical Sciences, Kerman, Iran.*; 2 *Researcher, Non-Communicable Diseases Research Center, Rafsanjan University of Medical Sciences, Rafsanjan, Iran. *; 3 *Assistant Professor of Medical Ethics, Faculty of Iranian Traditional Medicine, Kerman University of Medical Sciences, Kerman, Iran.*; 4 *Researcher, Nursing Research Center, Kerman University of Medical Sciences, Kerman, Iran.*

**Keywords:** Patients’ rights, Clinical ethics, Patients’ bill of rights.

## Abstract

Patients’ rights are among the most important criteria for evaluating the quality of health services. The current study aimed to determine the importance and observance of the patient's bill of rights.

This cross-sectional study was done in Kerman, Iran. The research samples were 217 patients and 204 personnel. The data collection tool was a researcher-made questionnaire in the scope of the patient's bill of rights, and data were analyzed by SPSS 15.

The results showed a significant difference between patients and the personnel on the subject of the patient's bill of rights and most of its dimensions (*P* < 0.01). However, no significant difference was found between their views on the observance of the patient's bill of rights and its dimensions. Also, 35.9% of patients as well as 25% of personnel considered the observance of patients’ rights unfavorable.

The participants were aware of the importance of the patient's bill of rights. It is necessary, however, to codify and approve the laws related to the rights of patients.

## Introduction

In recent years, societies have focused on individual and social ethics and rights, as well as the dignity of human beings. As a result, the concepts of patients’ rights and their support have been considered (1). The Universal Declaration of Human Rights in 1948 has defined patients’ rights based on the concept of equality of all human beings (2). Patients’ rights refer to specific legal privileges related to patients’ physical, psychological, spiritual and social needs that the healthcare system and the medical team observe them. (3, 4). On account of the increasing ethical challenges in medical care, policies have been devised to pay more attention to human rights, especially for patients as vulnerable individuals with significant needs (5). Healthcare providers must respect the patient more than ever (6), and in most countries throughout the world, they are required to observe some patients’ rights (7). Therefore, the role of professional healthcare is to protect people who cannot defend themselves. The patient's bill of rights (PBR) is a guide to choose and ensure the best future decisions for patients' benefits, and can be the starting point for full attention to patients' rights and a correct definition of the relationship between healthcare clients and providers (8). According to the laws of the American Medical Association, physicians must protect patients and promote their basic rights (9). The patient's bill of rights has been addressed in A lot of countries, so hospitals and healthcare facilities must observe it (10). In Iran, the Health Deputy at the Ministry of Health and Medical Education compiled the Patient’s Bill of Rights in 2002 and sent it to the subordinate organizations (11, 12).

The bill of rights includes the following topics: receiving proper services, patient's independence and decision-making rights, respect for the patient, privacy, and access to a complaints system. In some countries, the bill of rights also includes high-quality care, awareness of rights, pain relief, and easy death. (5). Patient's bill of rights creates a sense of security and increases satisfaction, while patient participation in therapeutic affairs reduces the cost of treatment and the length of hospital stay, and also prevents the occurrence of irreversible physical and emotional injuries (13, 14). Additionally, observing patients' rights will help improve and control their condition. Dissatisfied patients hardly follow the treatment orders and show fewer signs of recovery, and may leave or change the treatment center with an incomplete course of treatment (15). Therefore, non-compliance with patients' rights can endanger the health, life and safety of patients and weaken the relationship between healthcare personnel and patients, leading to less effective care and services to patients (16). Unfortunately, healthcare providers and clients do not have a proper relationship in many aspects of the patient's bill of rights (17). Studies have shown that in many cases, the patient's bill of rights is not implemented. According to these results (1, 18, 19), physicians, nurses and hospital managers did not have sufficient knowledge of patients’ rights, and these rights were not fully respected in the studied hospitals. In another study, despite the excellent knowledge of physicians, the results reported poor observance of the patients’ rights (20).

Given the importance of patients' rights and the different degrees of observance of the patient's bill of rights in different societies, the Iranian Ministry of Health has focused on the obsevance of the patient's bill of rights in recent years. Furthermore, few studies have been conducted on the importance and observance of the patient's bill of rights from the viewpoints of patients and healthcare personnel. The current study compared the importance and observance of the patient's bill of rights from the viewpoints of patients and healthcare personnel in Kerman. Therefore, the results can serve as a guide for authorities and professors at universities and improve patients’ condition, the quality of health care services, and the professional position of doctors and nurses.

## Method

This cross-sectional study was conducted in Kerman hospitals in 2018-2019. There are 4 governmental hospitals, 3 semi-governmental hospitals, and 2 private hospitals in Kerman, all of which were included in the research setting.

A cluster sampling method was used to collect data. Governmental, semi-governmental, and private hospitals were considered as a cluster, and sampling was then done according to the number of beds in each cluster. A pilot study with a sample size of 20 people (10 personnel and 10 patients) was carried out to estimate the sample size. The mean and standard deviation of observance of the patient's bill of rights from the viewpoints of personnel and patients were 2.84 ± 0.48, and 2.66 ± 0.72, respectively. 

The confidence coefficient was 95%, with the confidence range of 1.96, and type II error was 20% (0.84). Therefore, the sample size for each group was estimated to be 136, which, according to the cluster sampling method, was multiplied by 1.5. Finally, the sample size for each group was 204.

The study population consisted of patients admitted to hospitals in Kerman, aged ≥16, who were mentally and physically capable of answering questions, as well as the healthcare staff (nurses, midwives and doctors). The researchers started cluster sampling after obtaining permission from the Kerman University of Medical Sciences, and explaining their goals to the participants. A self-report questionnaire was used to collect information. In the case of illiterate patients, the researcher interviewed them and completed the questionnaire for them.

A two-part questionnaire was used to collect information. The first part of the questionnaire collected demographic information, including: 1) age, sex, marital status, level of education, occupation and income level for the patients, and 2) age, sex, marital status, level of education, occupation, type of employment and work experience for the staff (nurses, midwives, and doctors). The second part, a researcher-made questionnaire, was related to the ethics in the area of the patient's bill of rights and was completed by the patients and the staff. Therefore, focusing on the patient's bill of rights, the researchers formulated a questionnaire containing 20 items in 5 areas: "receiving optimal healthcare services" (9 items); "information provision" (4 items); "free decision-making" (2 items); "privacy and confidentiality" (2 items); and "access to an effective complaints handling system" (3 items). Each statement of the questionnaire was measured based on a two-point Likert scale. The significance of each phrase was measured using a 5-point Likert scale (very important = 4, important = 3, somewhat important = 2, not important = 1, and I have no idea = 0). The observance rate of each statement was also measured using a 5-point Likert scale (always observed = 4, often observed = 3, sometimes observed = 2, rarely observed = 1, and not observed at all = 0). The mean score was used to calculate scores. Therefore, the score range of significance and observance rate of the patient's bill of rights varied from 0 to 4, with higher scores indicating more important ethics and better observance. Also, in the area of observance of patients’ rights, mean scores of 0 - 1.34, 1.35 - 2.67 and above 2.67 were rather unfavorable, undesirable, and desirable, respectively. To determine the validity of this questionnaire, 5 experts in the field of medical ethics reviewed it, and their opinions were used to complete and correct the items. Internal consistency was used for reliability. The questionnaires were then distributed to 20 target people (10 patients and ten personnel), and Cronbach's alpha was calculated. The Cronbach's alpha values for the importance and observance of the patient's bill of rights were 0.88 and 0.81 from the staff’s point of view, and 0.87 and 0.85 from the patients’ point of view.

***Data Analysis***

SPSS 15 was used to analyze the data. Descriptive statistics (frequency, percentage, mean, and standard deviation) was used to describe the characteristics of the participants. Since the importance and observance scores of the patient's bill of rights were not within the normal distribution range in either group, non-parametric inferential statistics was used (Mann-Whitney U and Spearman correlation coefficient).

***Ethical Considerations***

The permission and a written letter of introduction were obtained from the Research Dean of Kerman University of Medical Sciences and the Ethics Committee of Afzalipour Hospital (IR.KMU.REC.1395.11) during data collection. The researchers fully respected the principles of data confidentiality.

## Results

A total of 217 patients and 204 healthcare personnel participated in this study. The demographic characteristics of patients and healthcare personnel were reported in Tables 1 and 2, respectively. The mean age of the patients was 43.88 ± 18.5, and the mean age of the healthcare personnel was 30.1 ± 6.92.

**Table 1:** Demographic characteristics of patients (n = 217)

Variable	Frequency	Valid Percent
**Healthcare provider centers ** Private hospitals Semi-governmental hospitals Governmental hospitals	41 27 149	18.9 12.4 68.7
**Age (year)** Less than 31 Between 31 - 40 Between 41 - 50 Between 51 - 60 Over 60	68 34 27 30 45	33.3 16.7 13.2 14.7 22.1
**Sex ** Male Female	89 125	41.6 58.4
**Marital status ** Single Married Other	36 168 12	16.7 77.8 5.5
**Education ** Illiterate Reading & writing literacy Diploma College education	28 46 81 51	13.6 22.3 39.3 24.8
**Job** Housewife/unemployed Employee Self-employed Retired	112 20 48 23	55.2 9.9 23.6 11.3

**Table 2:** Demographic characteristics of personnel (n = 204)

Variable	Frequency	Valid percent
**Place of service** Private hospitals Semi-governmental hospitals Governmental hospitals	42 25 137	20.6 12.2 67.2
**Age (year)** Less than 31 Between 31 - 40 Over 40	103 62 15	57.2 34.4 8.4
**Sex ** Male Female	31 164	15.9 84.1
**Marital status ** Single Married Other	70 124 4	35.4 62.6 2
**Education ** Diploma Associate degree BS*. MS**. PhD*** Specialized degree	24 3 153 7 13 3	11.8 1.5 75.4 3.4 6.4 1.5
**Profession ** Midwife Nurse Doctor	29 157 16	14.4 77.7 7.9
**Type of employment ** Permanent Temporary-to permanent Contractual Human source plan	38 29 52 67	20.4 15.6 28 36
**Work experience (year)** Less than/equal to 5 Between 6 and 10 Over 10	90 35 39	54.9 21.3 23.8

* *Bachelor of Science*

** *Master of Science*

*** *Philosophy Docto*r

The table 3 shows the importance and observance rate of the patients’ bill of rights from the view point of the study participants. According to table 3, a significant difference was found between the patients and the personnel in the importance of the patient's bill of rights and most of its dimensions, since patients' rights were more important to the patients. No significant difference was found between the patients and the personnel in the observance of the patient's bill of rights and its dimensions, except in "access to an effective complaints handling system". Also, 3.2% (n = 7) of the patients considered observance of patients’ rights rather unfavorable, 32.7% (n = 71) unfavorable, and 64.1% (n = 139) desirable. One percent (n = 2) of the personnel considered observance of patients’ rights very undesirable, 24% (n = 49) unfavorable, and 75% (n = 153) desirable.

**Table 3:** The importance and observance rate of the patient's bill of rights and its dimensions from the viewpoints of all participants

Patient's Bill of Rights	Importance Rate				Observance Rate			
	**Patients** **(mean & ** **standard ** **deviation)**	**Personnel** **(mean & ** **standard ** **deviation)**	**Test ** **Statistic**	***P***	**Patients** **(mean & ** **standard ** **deviation)**	**Personnel** **(mean & ** **standard ** **deviation)**	**Test ** **Statistic**	***P***
**Total Score**	3.56±0.44	3.47±0.42	- 2.52	0.01	2.91±0.74	3.02±0.57	- 1.3	0.19
**Receiving ** **Optimal ** **Healthcare ** **Services**	3.65±0.42	3.61±0.37	- 1.93	0.05	3.11±0.72	3.09±0.59	- 0.99	0.33
**Information ** **Provision **	3.52±0.61	3.39±0.57	- 3.08	0.002	2.81±0.98	2.92±0.75	- 0.45	0.65
**Free Decision-** **Making**	3.33±0.84	3.25±0.81	- 1.46	0.14	2.68±1.21	2.73±1	- 0.33	0.82
**Privacy & ** **Confidentiality**	3.65±0.62	3.39±0.63	- 5.58	< 0.001	3.21±0.79	3.19±0.73	- 0.66	0.51
**Access to a ** **Complaints ** **Handling System**	3.44±0.85	3.39±0.7	- 2.07	0.04	2.37±1.19	3.01±0.83	- 5.52	<0.001
**Mann-Whitney U**								

The perspectives of study participants about the importance and observance rate of ethical case law of the patients’ bill of rights were presented in table 4 and 5. According to table 4 and 5, in terms of the importance of the patient's bill of rights, the mean score of all items was higher than three from the viewpoints of all participants (minimum score of 0 and maximum score of 4). In the area of observance of the patient's bill of rights from the viewpoint of patients, the highest average scores were related to the items "privacy during treatment and confidentiality" (3.11), "respecting patients and being kind to them" (3.07), "skills of the healthcare team" (3.05), and "respecting the patient’s beliefs and religious and cultural values" (3.02). Also, in the area of observance of the patient's bill of rights from the patient's point of view, the lowest average scores were related to the items "compensation for medical errors" (2.05), "how to handle patient's complaints" (2.18), and "patient's right to complain" (2.4). From the viewpoints of patients, the mean scores of 16 items were less than 3, while from the viewpoints of personnel, only eight items were lower than 3.

**Table 4:** The importance and observance rate of ethical case law of the patient's bill of rights in dimension receiving optional healthcare service from the perspectives of patients and health care personnel

Case Laws of Patient's Bill of Rights		Importance Rate			Observance Rate		
		**Patients**	**Personnel**	***P***	**Patients**	**Personnel**	***P***
Receiving optimal healthcare services	Respecting patients and being kind to them	3.81±0.52	3.81±0.42	0.40	3.07±1.03	3.32±0.59	0.14
	Respecting the patients’ beliefs and religious and cultural values	3.36± 1.02	3.62±0.53	0.11	3.02±0.99	3.32±.64	0.005
	Being honest with patients	3.62±0.75	3.5±0.75	0.004	2.95±1.06	3.14±.73	0.28
	Non- discrimination among patients for healthcare services	3.48±1.0	3.69±0.7	0.05	2.75±1.22	3.19±0.84	0.001
	The skills of the healthcare team	3.69±0.76	3.82±0.4	0.3	3.05±0.93	3.26±0.77	0.03
	Paying attention to health, therapeutic and emotional benefits of the patients when providing services	3.66±0.68	3.53±0.7	0.01	2.98±1.03	3.18±0.73	0.15
	Providing well- being facilities	3.58±0.79	3.44±0.66	0.001	2.57±1.28	2.67±1.01	0.84
	Respecting patients’ time and providing fast services	3.7±0.67	3.49±0.64	0.001	2.62±1.28	2.96±0.96	0.02
	Paying attention to the needs of dying patients and their families	3.6±0.78	3.67±0.64	0.54	2.61±1.25	3.11±0.81	0.001

**Table 5:** The importance and observance rate of other dimensions ethical case law of the patient's bill of rights from the perspectives of patients and health care personnel

Case Laws of Patient's Bill of Right		Importance Rate			Observance Rate		
		**Patients**	**Personnel**	***P***	**Patients**	**Personnel**	***P***
Information provision	Providing information about treatment costs and insurance codes	3.57±0.76	3.3±0.76	0.001	2.77±1.18	2.92±0.86	0.76
	Introduction of medical personnel to patients	3.36±0.89	3.47±0.7	0.42	2.63±1.24	3.15±0.9	0.001
	Providing adequate information about diagnostic methods and therapeutic measures	3.55±0.77	3.53±0.69	0.38	2.71±1.18	2.96±0.88	0.10
	Providing information about how to access the doctor	3.52±0.84	3.41±0.67	0.00	2.47±1.3	2.96±0.92	0.001
Free decision making and selection	Freedom in selecting physicians	3.51±0.84	3.29±0.91	0.002	2.51±1.36	2.6±1.18	0.88
	The right to accept or reject treatments or therapeutic offers	3.17±1.12	3.26±0.97	0.82	2.65±1.21	2.94±1.02	0.02
Privacy and confidentiality	Privacy during treatment and confidentiality	3.63±0.83	3.75±0.55	0.25	3.11±0.97	3.35±0.81	0.001
	The right to having visitors	3.54±0.8	3.04±0.96	0.001	2.92±1.16	3.09±0.94	0.31
Access to an effective complaints handling system	Patients’ right to complain	3.35±0.98	3.38±094	0.71	2.4±1.32	3.24±0.84	0.001
	How to handle patients’ complaints	3.36±1.06	3.39±0.18	0.22	2.18±1.33	3.13±0.99	0.001
	Compensation for medical errors	3.66±0.84	3.53±0.75	0.001	2.05±1.5	2.88±1.07	0.001

## Discussion

The current study compared the importance and observance of the patient's bill of rights from the viewpoints of patients and staff in hospitals of Kerman. The results showed a significant difference between patients and personnel in terms of the importance of the patient's bill of rights and most of its dimensions, as it was found to be more important to patients than personnel. A statistically significant difference was found between patients and personnel in "access to an effective complaints handling system". Also, 35.9% of the patients and 25% of the personnel considered the observance of the patients’ rights very undesirable or unfavorable.

In the present study, the personnel and patients’ mean scores of the importance of patients’ rights were above three. The great importance of the patient's bill of rights to patients could be due to the fact that based on accreditation, hospitals are required to hold annual courses on patients' rights for personnel, and they have to complete them (21). Also, a study conducted in Izmir on physicians' awareness of the patients’ rights showed that 40% of the physicians were not aware of the patients' rights guidelines, and 63% of the participants did not even read them (3). According to Rashtabadi's study, nurses' attitudes toward patients’ rights will have a positive effect on their performance (22). In the present study, patients' rights were more important to patients than personnel, and since they are the healthcare recipients, the personnel must observe their rights. 

The present study showed that from nurses' and patients' viewpoints, the most important aspect of the patient's bill of rights is receiving optimal healthcare services. This has also been demonstrated in a study by Bazmi et al. (23). Doctors and nurses in hospitals are expected to provide healthcare services for patients; therefore, favorable healthcare services are the most important for patients (24).

In this study, the total score for the observance rate of the patient's bill of rights was not good from the viewpoints of the patients and the staff. Various studies have been carried out in Iran, indicating undesirable observance rates of the patient's bill of rights (25, 26). Studies have shown that 55.56% of the doctors in Saudi Arabia (27), 50% of the doctors and nurses in Egypt (28), 51% of the nurses and midwives in Turkey (29), and 31.2% of healthcare staff in Finland were not aware of the patient's bill of rights (30). Studies in different cities of Iran such as Yazd, Shiraz and Hamedan have reported observance of patients' rights to vary between 50.2% and 53.2% from the viewpoints of nurses and the healthcare team (31,32). Kazemnezhad and Hesamzadeh reported that the observance of patients' rights from the viewpoints of physicians and nurses was 33.3% at the poor level and 49.6% at the average level (18). Basiri Moghadam et al. in Gonabad (2011) showed that although the patients and staff had a good knowledge of the patient's bill of rights, the observance rate was not optimal (33).

A study in Uganda found that at least 36.5% of the patients encountered a challenge when claiming their rights, while 81.5% did not know anything about the patient's bill of rights (34). Also, 92.8% of patients in Sudan (35), and 75 % of patients and their companions in Egypt had no information about the patient's bill of rights (29). In a study by Nekoei Moghaddam et al., despite the desirable knowledge of patients and nurses (81.5%), the rate of observance of the patient's bill of rights was not favorable (66.92%) (25). In Babamahmoodi et al. study in Mazandaran, the lowest observance rate of the patient's bill of rights from patients' viewpoints was 14.59 (36).

Although the observance of the patient's bill of rights has been highlighted in Iranian hospitals, the performance of healthcare personnel must be changed, and health managers must emphasize the observance of the patient's bill of rights in hospitals.

From the viewpoints of staff and patients, the highest score in the observance of the patient's bill of rights was related to privacy and confidentiality. Humayun et al. showed that privacy and confidentiality were rarely observed in hospitals, and staff needed more training in observing ethics and patient privacy (37). The lowest score in the observance of the patient's bill of rights was related to free decision-making. According to this study, patients cannot freely choose their physicians, and the patient's right to either accept or reject proposed treatments was not scored favorably. Sultan Ahmadi et al. evaluated the rights of pregnant women in Kerman and obtained the lowest mean score in the area of decision-making (38). Therefore, appropriate action should be taken to ensure that patients can enjoy this right. Meanwhile, the low number of doctors may be one of the reasons why this has not been the case.

Patients may be free to accept the proposed treatment, but they are unlikely to be aware of this right, and the information needs to be provided for patients. In the present study, a statistically significant difference was found between the viewpoints of patients and personnel on accessing an effective complaints handling system and compensation for medical errors. According to patients, these two areas were often not observed. Studies have shown that access to an effective complaints handling system is important for patients (39,40). Friele and Sluijs showed that although only 7% of patients requested financial compensation, most patients expected impartial judgments about complaints in hospitals (39).

The current study had some limitations. This article cannot be generalized because of the cultural difference between Iranians and people in other countries, as the expectations of patients and personnel are different in various countries.

## Conclusion

In the present study, the importance of the patient's bill of rights was above moderate for both patients and personnel in Kerman. Also, a statistically significant difference was found between patients and personnel in the observance rate of the patient's bill of rights. The score of the observance of the patient's bill of rights was higher from the personnel's point of view compared to the patients' views, but not satisfactory in general. The current study can be used clinically and help hospital managers and macro managers to take steps and enforce laws to better comply with the patient's bill of rights. The results can also be used to train nurses because they showed that the patient's bill of rights was observed from the personnel's perspective, but not from the patients' viewpoint.

## Notes:

**Citation to this article:**

Dehghan M, Mehdipour-Rabori R, Rayani M, Zakeri MA, Mobasher M, Iranmanesh M, Rezai N. Comparison of the importance and observance of the patient's bill of rights from the perspectives of patients and personnel of hospitals in Kerman. J Med Ethics Hist Med. 2020; 13: 5.
